# Interferon-gamma release assays for diagnosis of latent TB infection in chronic kidney diseases and dialysis patients

**DOI:** 10.3389/fcimb.2022.1046373

**Published:** 2022-11-14

**Authors:** Pattorn Hayuk, Sarinya Boongird, Prapaporn Pornsuriyasak, Jackrapong Bruminhent

**Affiliations:** ^1^ Department of Medicine, Faculty of Medicine Ramathibodi Hospital, Mahidol University, Bangkok, Thailand; ^2^ Division of Nephrology, Department of Medicine, Faculty of Medicine Ramathibodi Hospital, Mahidol University, Bangkok, Thailand; ^3^ Division of Pulmonary and Critical Care, Department of Medicine, Faculty of Medicine Ramathibodi Hospital, Mahidol University, Bangkok, Thailand; ^4^ Division of Infectious Diseases, Department of Medicine, Faculty of Medicine Ramathibodi Hospital, Mahidol University, Bangkok, Thailand

**Keywords:** *Mycobacterium tuberculosis*, cell-mediated immunity, peritoneal dialysis, hemodialysis, end-stage kidney disease

## Abstract

**Introduction:**

Patients with chronic kidney disease (CKD), especially end-stage kidney disease (ESKD), are at risk of developing tuberculosis (TB). The prevalence and predictors of LTBI assessed by a high-sensitivity, high-specificity test such as an interferon-gamma release assay (IGRA) has not been thoroughly explored.

**Methods:**

All patients with CKD were prospectively recruited from September 2020 to November 2021 and retrospectively reviewed from December 2020 to November 2021. The prevalence of LTBI was determined using IGRA by CKD stage and dialysis type. Predictors of LTBI were assessed by logistic regression analysis.

**Results:**

In total, 199 patients with CKD were enrolled (102 prospectively, 97 retrospectively). Of these, 173 patients were evaluable (mean age, 53 ± 16 years; 44% male). Ninety-five (55%) patients had ESKD and were maintained on renal replacement therapy. Overall, 39 (22.5%) patients had LTBI with a prevalence of 25.0%, 12.5%, 25.0%, 25.0%, and 24.2% among patients with CKD stage 1, 2, 3a, 3b, and ESKD, respectively (*p*=0.89). Among patients with ESKD, the prevalence of LTBI was higher in those on hemodialysis than in those on peritoneal dialysis (28.9% vs. 5.3%, *p*=0.03). In the multivariable analysis of patients with ESKD, drinking alcohol was significantly associated with LTBI (odds ratio, 8.51; 95% confidence interval, 1.24–58.38; *p*=0.029), and hemodialysis was marginally associated with LTBI (odds ratio, 8.14; 95% confidence interval, 0.95–69.91; *p*=0.056).

**Conclusion:**

In TB-endemic settings, 20% of patients with CKD and 25% of patients with ESKD may have LTBI. Alcohol consumption and hemodialysis can help to identify high-risk patients with ESKD and potentially screen for LBTI.

## Introduction

Tuberculosis (TB) is a worldwide infectious disease with high morbidity and mortality, and the number of patients with TB infection reached 10 million globally in 2019 (Kanchar and Swaminathan, 2019). The incidence of TB is considered to be higher among patients with chronic kidney disease (CKD) than in the general population. Patients with CKD are at increased risk of TB because of their impairment in cell-mediated immunity, and those who were latently infected with TB in the past are prone to develop TB reactivation (Al-Efraij et al., 2015). The risk of developing active TB is 8 to 25 times higher in patients with CKD than in the general population; their TB-associated mortality rate is also higher, especially those with end-stage kidney disease (ESKD) requiring renal replacement therapy (RRT) (Lundin et al., 1979; Dobler et al., 2011). Furthermore, TB diagnosis in patients with CKD is usually delayed because of atypical and occasional extrapulmonary manifestations (Vikrant, 2019).

Because of their relatively immunosuppressed condition, reactivation of latent TB infection (LTBI) is the core pathogenesis in this patient population. In particular, patients with ESKD who are maintained on hemodialysis (HD) are more likely to have a higher prevalence of LTBI than those maintained on peritoneal dialysis (PD) because of their more frequent hospital visits and longer hospital stays. Therefore, identifying patients with LTBI and preventing reactivation of active TB are essential in this population.

The tuberculin skin test (TST) has been widely used to determine the LTBI status in the general population and in patients with CKD. However, the low sensitivity of this test may limit its use in patients with CKD because of their decreased T-cell function (Smirnoff, 1998). Furthermore, the TST is less specific to *Mycobacterium tuberculosis*, especially in patients who have received the bacillus Calmette–Guérin (BCG) vaccine. Interferon-gamma release assays (IGRAs), either by enzyme-linked immunosorbent spot (ELISpot) or enzyme-linked immunosorbent assay (ELISA), are being increasingly used to detect cases of LTBI. IGRAs have been proven helpful in immunocompromised patients with T-cell dysfunction, particularly those who have received the BCG vaccine (Brock et al., 2004; Simsek et al., 2010).

Studies focusing on the rate of LTBI among patients with CKD in Thailand are limited, and most such studies used the TST. Therefore, we analyzed the epidemiology of LTBI in Thai patients with CKD (especially those with ESKD requiring RRT) in TB-endemic areas using a highly sensitive and specific test (IGRA). Additionally, we investigated risk factors that may assist clinicians in identifying patients at risk of LTBI in an effort to develop prevention strategies for this vulnerable population.

Overall, the aim of this study was to elucidate the prevalence and predictors of LTBI among Thai patients with CKD, including those with ESKD receiving RRT by either HD or PD.

## Methods

### Study population

All patients with CKD who underwent an investigation of their LTBI status at Ramathibodi Hospital were prospectively recruited from September 2020 to November 2021 and retrospectively reviewed from December 2020 to November 2021.

LTBI was defined as a positive IGRA result without clinical or radiographic findings compatible with active TB. The T-SPOT^®^.TB assay (Oxford Immunotec, Abingdon, UK) was performed, and the interferon-gamma level of the post-reaction supernatant was then measured by ELISpot. The results were interpreted as positive, negative, borderline, or indeterminate (Dyrhol-Riise et al., 2010; Banach and Harris, 2011). Patients with borderline results were included in a positive test for comparative analysis. Those with indeterminate results were excluded from the study.

In the evaluation of radiographic findings, a lung lesion compatible with active TB was defined as a new patch of consolidation, collapse, lymphadenopathy, mass or nodule, or cavitary lesion without other proven etiology. Prior TB was radiographically defined as the presence of fibrotic infiltrates with pleural thickening or calcified nodules over the upper lung fields along with other fibrotic lesions documented from previous TB disease (Jasmer et al., 2000). A certified radiologist interpreted all chest radiographs in this study.

>CKD is defined by the presence of kidney damage or decreased kidney function for at least 3 months, irrespective of the cause. Kidney damage generally refers to pathologic anomalies in the native or transplanted kidney as established *via* imaging or biopsy or as deduced from clinical markers such as increased albuminuria (albumin-to-creatinine ratio of >30 mg/g) or urinary sediment alterations. Decreased kidney function refers to a reduced glomerular filtration rate (<60 mL/min/1.73 m^2^) (Navaneethan et al., 2021). The prevalence of LTBI was determined by the CKD stage, which was classified by the estimated glomerular filtration rate (stage 1, 2, 3a, 3b, 4, 5, or 5d). Patients with stage 5d CKD were those who were maintained on RRT by either HD or PD. The dialysis mode was defined as its use in the past 3 months prior to the T-SPOT^®^.TB test. Every HD session regularly lasted for 4 hours in accordance with the National Kidney Foundation Kidney Disease Outcome Quality Initiative ([[NoAuthor]]) with two to three sessions per week depending on the patient’s residual renal function and adequacy of dialysis.

Current smokers were defined as those who had smoked within 3 months prior to the study (Lin et al., 2009). Current alcohol drinkers were defined as women who had drunk more than 7 standard alcoholic drinks and men who had drunk more than 14 standard drinks within 3 months prior to the study.

### Data collection

The following demographic and clinical data were recorded in a standardized case report form: age, sex, weight, body mass index (BMI), etiology of CKD, underlying comorbidities, history of TB, history of BCG vaccination, respiratory and constitutional symptoms, smoking status, alcohol drinking status, blood hemoglobin concentration, total white blood cell count, absolute lymphocyte count, serum albumin concentration, blood urea nitrogen concentration, and creatinine concentration.

### Statistical analysis

The patients were classified according to their LTBI status for further comparison. Intergroup differences were analyzed using Student’s t-test for numerical variables and the chi-square test for categorical variables. Predictors of LTBI were assessed by logistic regression analysis. Multivariable analysis was used to identify factors associated with the LTBI status. All potential predictors with a *p* value of <0.10 were included in the stepwise variable selection procedure. A two-sided *p* value of <0.05 was considered statistically significant. All analyses were performed using SPSS Version 13.0 (SPSS Inc., Chicago, IL, USA).

## Results

### CKD population

**Figure 1:**
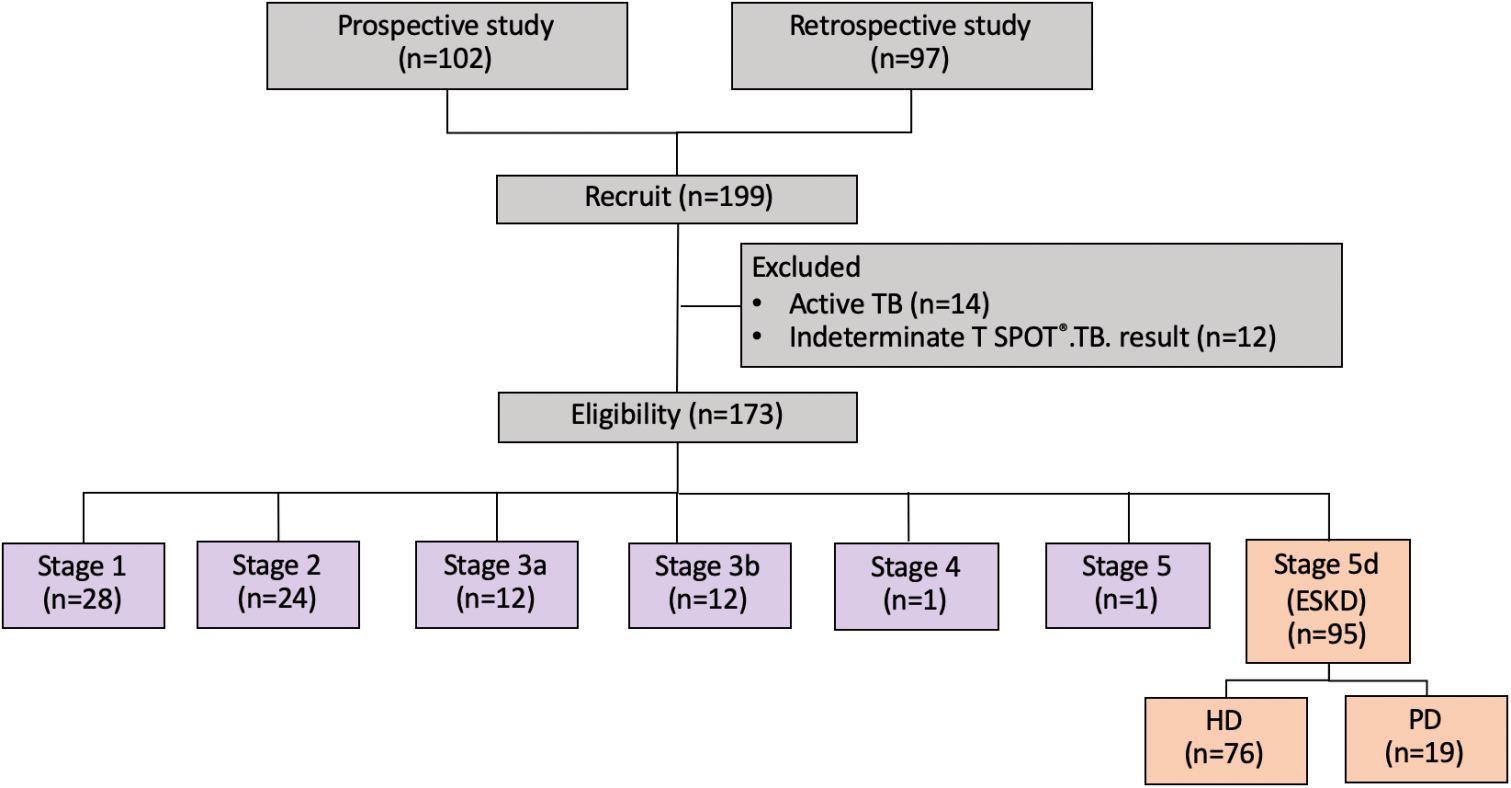
Study flow chart. ESKD, end-stage kidney disease; HD, hemodialysis; PD, peritoneal dialysis.

The data of 199 patients with CKD were retrieved, among whom 97 patients were from the Ramathibodi Hospital database. Moreover, 102 patients were prospectively identified during the study period **(** **Figure 1** **).** Twenty-six patients were excluded because of active TB infection (n=14) and indeterminate T-SPOT^®^.TB results (n=12). Therefore, 173 patients were eligible for the analysis. A total of 16.2%, 13.9%, 6.9%, 6.9%, 0.6%, 0.6%, and 54.9% of the patients had underlying stage 1, 2, 3a, 3b, 4, 5, and 5d CKD, respectively. The patients’ mean ± standard deviation age was 52.4 ± 15.8 years. A total of 43.3% of the patients were male, and the mean BMI was 23.56 ± 4.31 kg/m^2^. The etiologies of CKD were diabetes mellitus (4.62%), hypertension (1.15%), chronic glomerulonephritis (8.67%), other conditions (2.89%), and unknown (38.15%). The comorbidities included diabetes mellitus (18.49%), hypertension (66.47%), atherosclerotic vascular disease (7.51%), and human immunodeficiency virus (HIV) infection (0.50%). Among all patients with CKD, 29.48% received immunosuppressants, 82.66% received BCG vaccination, 6.35% reported being a current smoker, and 4.62% reported alcohol consumption **(** **Table 1** **)**.

**Table 1:** Characteristics of patients with CKD.

Characteristics	Total (n=173)	LTBI (n=39)	Non-LTBI (n=134)	*p* value
Age, years	52.43 ± 15.81	54.08 ± 15.77	51.96 ± 15.84	0.462
Male sex	75 (43.35)	17 (43.58)	58 (43.28)	0.973
BMI, kg/m^2^	23.56 ± 4.31	23.25 ± 3.96	23.65 ± 4.41	0.619
Cause of CKD				0.735
Diabetes mellitus	8 (4.62)	2 (5.12)	6 (4.47)	
Hypertension	2 (1.15)	0 (0.00)	2 (1.49)	
Chronic glomerulonephritis	15 (8.67)	2 (5.13)	13 (9.70)	
Others	5 (2.89)	1 (2.56)	4 (2.98)	
Unknown	143 (82.65)	39 (10.40)	137 (77.45)	
Comorbidities				
Diabetes mellitus	32 (18.49)	6 (15.38)	26 (19.40)	0.569
Hypertension	115 (66.47)	26 (66.67)	89 (66.41)	0.977
ASCVD	13 (7.51)	2 (5.12)	11 (8.20)	0.514
HIV infection	1 (0.50)	0 (0.00)	1 (0.50)	0.594
Receiving immunosuppressants	51 (29.48)	9 (23.07)	42 (31.34)	0.319
BCG vaccination	143 (82.66)	31 (79.48)	112 (83.58)	0.552
History of TB infection	7 (4.04)	1 (2.56)	6 (4.47)	0.558
Current smoking	11 (6.35)	4 (10.25)	7 (5.22)	0.257
Current alcohol drinking	8 (4.62)	4 (10.25)	4 (2.98)	0.051
CKD stage				0.89
1	28 (16.20)	7 (25.00)	21 (75.00)	
2	24 (13.90)	3 (12.50)	21 (87.50)	
3a	12 (6.93)	3 (25.00)	9 (75.00)	
3b	12 (6.93)	3 (25.00)	9 (75.00)	
4	1 (0.57)	0 (0.00)	1 (100)	
5	1 (0.57)	0 (0.00)	1 (100)	
5d (ESKD)	95 (54.90)	23 (24.20)	72 (75.80)	
White blood cell count, cells/mm^3^	6712 ± 2150	7031 ± 2902	6840 ± 2211	0.661
Neutrophils, %	69 ± 12	67 ± 12	66 ± 13	0.649
Lymphocytes, %	18 ± 8	22 ± 10	22 ± 11	0.886
Absolute lymphocyte count, cells/mm^3^	1149 ± 557	1569 ± 1028	1490 ± 918	0.649
Hemoglobin, g/dL	11 ± 1.7	11.15 ± 2.0	11.24 ± 1.7	0.781
Albumin, g/L	3.7 ± 0.57	3.7 ± 0.57	3.7 ± 0.52	0.959
Parathyroid hormone, pg/mm^3^	579 ± 494	607 ± 425	570 ± 517	0.761
Calcium, mg/dL	9.3 ± 1.0	9.4 ± 0.9	9.3 ± 1.1	0.667
Phosphorus, mg/dL	4.7 ± 1.4	4.4 ± 1.4	4.8 ± 1.4	0.242
Blood urea nitrogen, mg/dL	44.6 ± 19.2	33.8 ± 15.0	37.0 ± 22.0	0.410
Creatinine, mg/dL	9.2 ± 3.5	5.45 ± 4.4	5.65 ± 5.1	0.823

Data are presented as mean ± standard deviation or n (%).

ASCVD, atherosclerotic cardiovascular disease; BCG, bacillus Calmette–Guérin; BMI, body mass index; CKD, chronic kidney disease; ESKD, end-stage kidney disease; HIV, human immunodeficiency virus; LTBI, latent tuberculosis infection; TB, tuberculosis.

**Figure 2:**
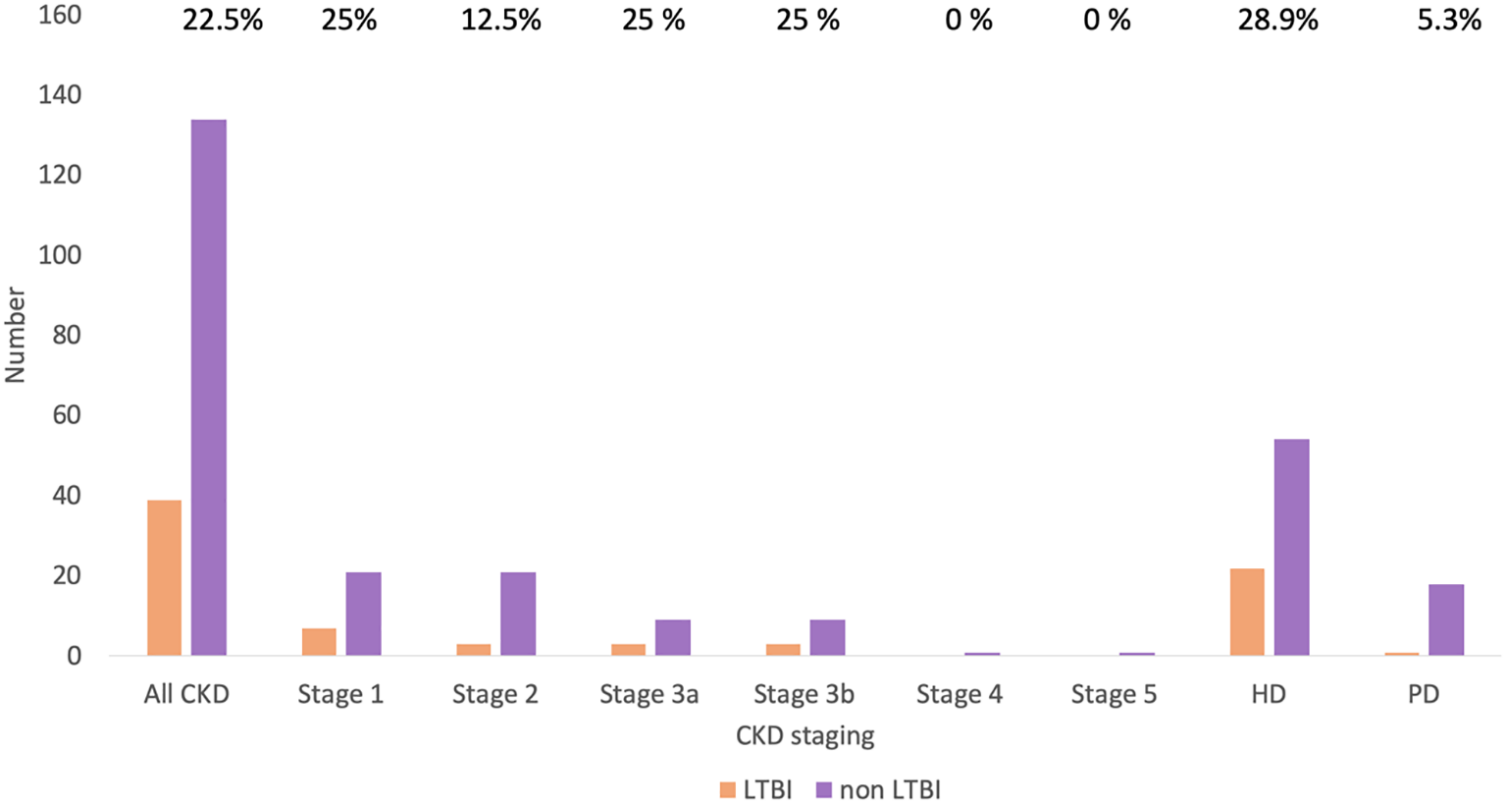
Prevalence of LTBI in patients with CKD by CKD stage. CKD, chronic kidney disease; HD, hemodialysis; LTBI, latent tuberculosis infection; PD, peritoneal dialysis.

The prevalence of LTBI among patients with CKD was 22.5%. The distribution of LTBI was 25.0%, 12.5%, 25.0%, 25.0%, and 24.2% among patients with underlying stage 1, 2, 3a, 3b, and 5d CKD, respectively (*p*=0.89) **(** **Figure 2** **).** A comparison of the demographics and laboratory data of patients with and without LTBI is presented in **Table 1**. There were no significant differences in parameters between patients with and without LTBI.

### ESKD population

A total of 95 (55%) patients had ESKD requiring RRT. The characteristics of the patients with ESKD are presented in **Table 2**. In this subpopulation, 80% and 20% of patients were maintained on HD and PD, respectively. Their mean age was 44.30 ± 11.54 years. Approximately 58.95% were male, and the mean BMI was 23.01 ± 4.19 kg/m^2^. The etiologies of CKD were diabetes mellitus (8.42%), hypertension (2.10%), chronic glomerulonephritis (14.73%), other conditions (5.26%), and unknown (69.47%). The comorbidities comprised diabetes mellitus (14.73%), hypertension (89.47%), and atherosclerotic vascular disease (8.42%). Three (3.15%) and 94 (98.94%) patients had previously received immunosuppressants and BCG vaccination, respectively. Six (6.31%) patients reported having smoking and drinking habits each.

**Table 2:** Characteristics of patients with ESKD.

Characteristics	Total (n=95)	LTBI (n=23)	Non-LTBI (n=72)	*p* value
Age, years	44.30 ± 11.54	51.95 ± 15.84	54.08 ± 15.77	0.463
Male sex	56 (58.95)	13 (56.52)	43 (59.72)	0.786
BMI, kg/m^2^	23.01 ± 4.19	23.25 ± 3.96	23.64 ± 4.41	0.619
Cause of CKD				0.776
Diabetes mellitus	8 (8.42)	2 (8.69)	6 (8.33)	
Hypertension	2 (2.10)	0 (0.00)	2 (2.77)	
Chronic glomerulonephritis	14 (14.73)	2 (8.69)	12 (16.67)	
Others	5 (5.26)	1 (4.34)	4 (5.55)	
Unknown	66 (69.47)	18 (78.26)	48 (66.67)	
Comorbidities				
Diabetes mellitus	14 (14.73)	2 (8.69)	12 (16.67)	0.348
Hypertension	85 (89.47)	20 (86.95)	65 (90.27)	0.651
ASCVD	8 (8.42)	1 (4.34)	7 (9.72)	0.410
HIV	1 (1.05)	0 (0.00)	1 (1.38)	0.567
Receiving immunosuppressants	3 (3.15)	1 (4.34)	2 (2.77)	0.708
BCG vaccination	94 (98.94)	23 (1.00)	71 (98.61)	0.570
History of TB infection	3 (3.15)	0 (0.00)	3 (4.16)	0.320
Current smoking	6 (6.31)	3 (13.04)	3 (4.16)	0.128
Current alcohol drinking	6 (6.31)	4 (17.39)	2 (2.77)	0.012
Renal replacement therapy				0.031
Hemodialysis	76 (80.00)	22 (28.94)	54 (71.05)	
Peritoneal dialysis	19 (20.00)	1 (5.26)	18 (94.73)	

Data are presented as mean ± standard deviation or n (%).

ASCVD, atherosclerotic cardiovascular disease; BCG, bacillus Calmette–Guérin; BMI, body mass index; CKD, chronic kidney disease; ESKD, end-stage kidney disease; HIV, human immunodeficiency virus; LTBI, latent tuberculosis infection; TB, tuberculosis.

A total of 23 (24%) patients had LTBI. Among these patients, the prevalence of LTBI was significantly higher in those receiving HD than in those receiving PD (28.94% and 5.26%, respectively; *p*=0.031). A comparison of the variables of patients with and without LTBI is presented in **Table 2**. A significantly higher proportion of patients with ESKD reported using alcohol in the LTBI group than in the non-LTBI group (*p*=0.012). Furthermore, more patients were maintained on HD in the LTBI group than in the non-LTBI group (*p*=0.031).

In the multivariable analysis of patients with ESKD, drinking alcohol was significantly associated with LTBI (odds ratio, 8.51; 95% confidence interval, 1.24–58.38; *p*=0.029), and HD was marginally associated with LTBI (odds ratio, 8.14; 95% CI, 0.95–69.91; *p*=0.056) **(** **Table 3** **)**.

**Table 3:** Risk factors for LTBI among patients with ESKD by logistic regression analysis.

Risk factors	Univariable analysis		Multivariable analysis	
	OR (95% CI)	*p* value	OR (95% CI)	*p* value
Age	1.02 (0.98–1.06)	0.320		
Male sex	0.87 (0.34–2.27)	0.786		
BMI	0.91 (0.79–1.03)	0.154		
Diabetes mellitus	0.47 (0.09–2.30)	0.357		
Hypertension	0.72 (0.17–3.03)	0.653		
ASCVD	0.41 (0.05–3.57)	0.424		
Alcohol drinking	7.36 (1.25–43.32)	0.027	8.51 (1.24–58.38)	0.029
Receiving immunosuppressant	1.59 (0.14–18.39)	0.71		
Current smoking	3.45 (0.65–18.43)	0.148		
Hemodialysis	7.33 (0.92–58.33)	0.060	8.14 (0.95–69.91)	0.056

ASCVD, atherosclerotic cardiovascular disease; BMI, body mass index; CI, confidence interval; ESKD, end stage kidney disease; LTBI, latent tuberculosis infection; OR, odds ratio.

## Discussion

This study investigated the prevalence of LTBI using the T-SPOT^®^.TB assay as a diagnostic tool in Thai patients with CKD, focusing on the need for dialysis. Approximately 20% of the patients with CKD were found to have LTBI. More interestingly, approximately 25% of the patients requiring RRT also had LTBI. Among the latter, those who were current alcohol drinkers and were receiving HD were more likely to be diagnosed with LTBI.

The population of patients with CKD is increasing worldwide, and TB is a commonly associated infectious disease (Stevens et al., 2010; Hill and Fogarty, 2012). Investigation for LTBI is crucial to identify patients at risk of TB reactivation and offer them LTBI therapy. The IGRA-positive rate reportedly ranges from 21% to 40% in patients with CKD, whereas 6% to 11% of patients have indeterminate IGRA results (Lee et al., 2009; Lee et al., 2010). The prevalence of LTBI in the present study was approximately 22.5%. We propose that patients with CKD in Thailand have a much higher prevalence of LTBI than the general population and should be prioritized for targeted prevention of active TB disease, especially IGRA-positive patients.

Our study is unique regarding use of the IGRA, which offers several advantages over the TST in terms of convenience and accuracy [Brock, Weldingh et al., 2004, Simsek, Alpar et al., 2010]. Use of the TST is limited in Thai patients because of possible cross positivity with the BCG strain from childhood vaccination and an emerging prevalence of non-tuberculous mycobacterial disease in Asia (Yu et al., 1999; Lai et al., 2010). Furthermore, the ELISpot assay offers advantages over ELISA by incorporating a positive control (mitogen) tube and negative control (no antigen) tube; the immune reaction to *M. tuberculosis*-specific antigens can be differentiated from false-positive results due to nonspecific activation and false-negative results due to immunosuppression. Thus, the IGRA is a better screening test for LTBI than is the TST. Furthermore, ELISpot has higher sensitivity than ELISA for detection of LTBI in relatively immunocompromised patients. However, this does not help to differentiate LTBI from active TB disease. Two IGRAs are currently commercially available in Thailand: the QuantiFERON^®^-TB Gold In-Tube test and the T-SPOT^®^.TB test. Although not 100% precise, their sensitivity of 98.0% and specificity of 99.1% have been proven helpful in immunocompromised patients with T-cell dysfunction, particularly those who have received BCG vaccination (Brock et al., 2004; Simsek et al., 2010).

It has been previously assumed that because patients undergoing HD frequently visit the HD room, they are more likely to acquire TB *via* airborne transmission than are patients undergoing PD. However, a report of patients undergoing PD in Spain and Taiwan showed a comparable prevalence of LTBI (Palomar et al., 2011). This could be explained by the nature of dialysis settings and practices in each country. In contrast to a previous report (Banach and Harris, 2011), alcohol drinking was not identified as an independent risk factor for LTBI among the CKD population in the present study. Instead, consuming alcohol more than once a week is reportedly an independent predictor of LTBI in the general Asian population (Yap et al., 2018). If an IGRA is unavailable, the focus should be on current alcohol drinkers and HD recipients. Different combinations of these predictors may be helpful to select the target population for preventive therapy for LTBI. However, the costs and benefits of preventive therapy in this particular population should be further evaluated. Furthermore, risk factors for LTBI may vary based on the characteristics of patients in different studies, such as age, race, the prevalence of HIV infection, and the mode of dialysis. Further large-scale investigations are necessary to confirm this finding and investigate its possible causes.

The present study has several limitations. First, this study was conducted in a tertiary referral center that includes patients with more underlying comorbidities and a higher prevalence of LTBI than the general population. Second, recall bias in the retrospective part of the study may have limited our acquisition of the patients’ actual previous TB exposure. Third, the long time gap between the chest X-ray examination and the T-SPOT^®^.TB assay in some patients prevented assessment of the most current clinical condition. Fourth, the laboratory data were missing in a few patients. Fifth, a true prevalence of LTBI among non-dialysis CKD patients and dialysis patients may not be truly represented and subjected to selection bias since this LTBI screening is still not widely utilized in our resource-retrained setting. However, our data could encourage clinicians to better explore this particular latent infection among these high-risk populations. Finally, active TB infection cannot be completely ruled out based on subjective symptoms, and chest X-ray examination may not be sensitive enough to provide evidence of active TB infection.

In conclusion, approximately 20% of patients with CKD and 25% of patients with ESKD may have LTBI. Therefore, LTBI screening by highly specific and sensitive tests such as the IGRA is encouraged in Thailand. Among patients with ESKD, those who maintain HD for RRT and drink alcohol are at greater risk of LTBI. These two predictors may help to better identify high-risk patients with ESKD and potentially screen them for LBTI.

## Data availability statement

The raw data supporting the conclusions of this article will be made available by the authors, without undue reservation.

## Ethics statement

The studies involving human participants were reviewed and approved by Faculty of Medicine Ramathibodi Hospital, Mahidol University, Bangkok, Thailand. The patients/participants provided their written informed consent to participate in this study.

## Author contributions

Conceptualization: JB. Data collection: PH, JB. Data analysis: PH, JB. Manuscript writing (original draft): PH, JB. Manuscript reviewing and editing: PH, SB, PP, JB. All authors contributed to the article and approved the submitted version.

## Conflict of interest

The authors declare that the research was conducted in the absence of any commercial or financial relationships that could be construed as a potential conflict of interest.

## Publisher’s note

All claims expressed in this article are solely those of the authors and do not necessarily represent those of their affiliated organizations, or those of the publisher, the editors and the reviewers. Any product that may be evaluated in this article, or claim that may be made by its manufacturer, is not guaranteed or endorsed by the publisher.

## Abbreviations

CKD, chronic kidney disease; ESKD, end-stage kidney disease; TB, tuberculosis; LTBI, latent TB infection.
